# Microcystin: From Blooms to Brain Toxicity

**DOI:** 10.33696/Signaling.6.131

**Published:** 2025

**Authors:** Ethan Hedrick, Aryaman Tiwari, Suryakant Niture, Qing Cheng, Deepak Kumar, Somnath Mukhopadhyay

**Affiliations:** 1 Neuroscience Research Program, JLC- Biomedical Biotechnology Research Institute, North Carolina Central University, Durham, NC 27707, USA; 2 Pharmaceutical Sciences, North Carolina Central University, Durham, NC 27707, USA; 3 Department of Chemistry & Biochemistry, North Carolina Central University, Durham, NC 27707, USA

**Keywords:** Cyanotoxins, Microcystins, Neuro-cytotoxicity, Neurodegenerative diseases

## Abstract

An increase in the temperature of lakes and ponds facilitates the over-growth of photosynthetic cyanobacteria that produce a class of toxins called cyanotoxins. The abundance of cyanobacteria poses a significant threat to drinking and irrigation water supplies, and therefore, cyanotoxins have become a major class of environmental pollutants. Microcystins, the most common cyanotoxins, are cyclic peptides produced by cyanobacteria through non-ribosomal peptide synthases, and currently, approximately 279 microcystins have been identified to date. Exposure to microcystins can cause liver and brain cytotoxicity, dermatologic, gastrointestinal, respiratory, and neurologic signs and symptoms, and affect human health. Notably, microcystin-leucine arginine can breach the blood-brain barrier by the transporter proteins, organic anion transporting polypeptides, leading to neuroinflammation, and changes in neurocircuitry resulting in behavioral alterations. In this review, we provide an update of the current literature on the detrimental effects of microcystins on the brain, focusing on their potential role in Alzheimer’s and Parkinson’s diseases. We discuss the current findings along with the cellular mechanisms involved and provide a brief narrative of the scope of future studies, especially to address the effects of microcystins along with genetic and other risk factors (like alcohol and other drugs) on neurodegenerative disease.

## Introduction

Microcystins (MCs) are cyclic heptapeptides and toxic biomolecules produced by cyanobacteria (blue-green algae) that commonly grow in fresh bodies of water such as lakes, rivers, groundwater, and natural reservoirs. Cyanobacteria release several cyanotoxins in the aquatic system, including MCs, and more than 279 MCs have been identified so far. MC-LR is abundantly found in aquatic systems [1]. MC can contaminate drinking water and food supplies as well as bioaccumulate into crops and aquatic life, which are then consumed by humans [2]. Besides food and drinking water, people can be exposed to MC through dermal contact from swimming in contaminated water as well as inhaling aerosolized MC [3]. Prolonged, repeated exposure to MC can be toxic to the brain, liver, and other organs, increasing the risk of developing chronic illnesses [2]. MC’s effect on the brain has become a significant area of research due to its ability to cross the blood-brain barrier [4]. Additionally, intensifying cyanobacterial blooms along with warmer temperatures due to climate change can increase MC production in water bodies, thereby exacerbating the issue and its pertinence [5,6]. In this review, we will discuss the source of MC, why it has become a growing problem, and its effects on the brain.

## MC Source and Structure

MC is predominantly produced by cyanobacteria in the genus *Microcystis* [7]. However, it can be produced by other genera of cyanobacteria such as *Oscillatoria*, *Planktothrix*, *Nostoc*, *Dolichospermum*, *Anabaenopsis*, *Aphanizomenon*, *Aphanocapsa*, *Arthrospira*, *Cylindrospermum, Fischerella, Hapalosiphon, Phormidium, Raphidiopsis, Scytonema, Umezakia, and Woronichinia* [8]. Because there is a large variety of MC-producing cyanobacteria, scientists use different techniques to identify them. Light microscopy has been commonly utilized to identify MC-producing cyanobacteria. However, MC-producing cyanobacteria have varying morphologies within the same species, making it difficult to identify them with light microscopy [7]. More recently, real-time polymerase chain reaction (RT-PCR) has emerged as a more precise method for identifying MC-producing cyanobacteria. This technique allows for the detection and analysis of the expression of the microcystin synthetase genes [7]. This technique has been used to identify hot spots for MC-producing cyanobacteria globally.

**Figure 1.**
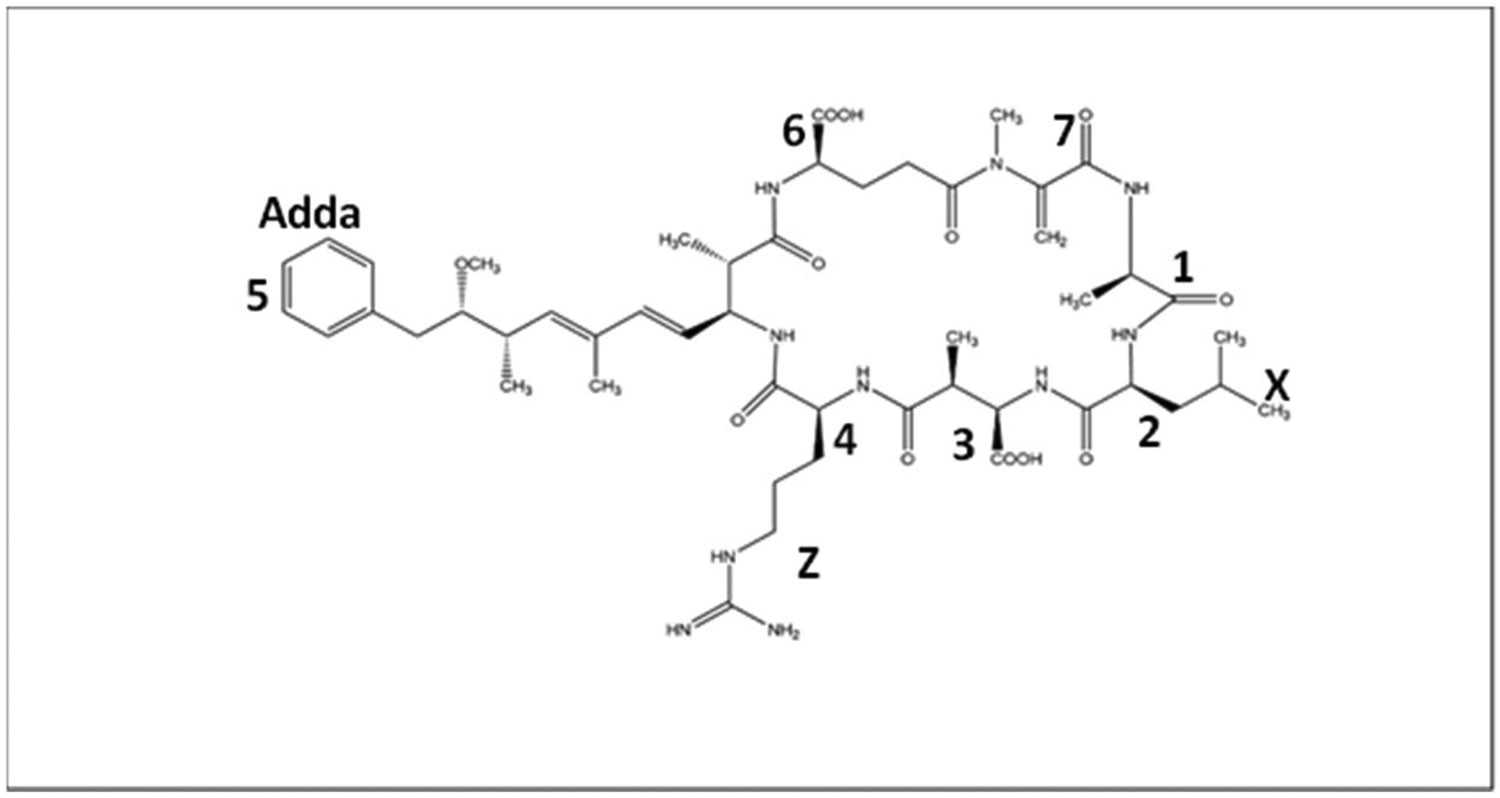
Structure of MC-LR where X and Z are Leucine and Arginine at the 2 and 4 positions, respectively.

Structurally, most MCs are cyclic heptapeptide molecules with the same basic structure of seven amino acids forming a ring with the Adda group and a β-amino acid at the end [1]. Its basic structure is cyclo-(D-Ala1-X2-D-Masp3-Z4-Adda5-D-γ-Glu6-Mdha7), with the second and fourth amino acids leading to the most variation (Figure 1) [1]. The Adda residue is believed to be the major cause of toxicity because it allows MC to bind to the enzymes protein phosphatases PP1 and PP2A, thereby inhibiting them [2]. The standard nomenclature for MC is MC-XZ, with X and Z being the second and fourthposition amino acids, respectively [1]. MC-LR (Figure 1) has been the most widely studied MC molecule due to its high toxicity and widespread occurrence worldwide in irrigation and drinking water [2].

## Environmental Temperature and MC Production

Climate change and global warming have brought MC research to the forefront. For example, optimum growth of *Microcystis* was found at temperatures between 20°C and 35°C [5]. Trung *et al.* examined cyanobacterial growth between 27°C and 37°C. They found that higher water temperatures resulted in higher growth rates of cyanobacteria but lower production rates of MC [6]. Conversely, Stark *et al.* showed that decreasing the temperature from 26°C to 19°C slowed the cell growth but increased the production of MC [9]. As a result, microcystic blooms are most toxic in the early spring and the least during mid-summer due to the higher production of MC at cooler temperatures [10]. Climate change has accelerated the melting of glaciers, resulting in rising ocean levels. Consequently, saltwater from the ocean has infiltrated bodies of freshwater, increasing their salinity through a process called saltwater intrusion [6]. Moreover, an increase in freshwater salinity can inhibit the growth of some *Microcystis*; however, some *Microcystis* are resistant to increasing salinity [6]. Trung *et al.* found that *Microcystis* growth increased when salinity was raised from 0.5% to 12% salinity (constant temperature maintained), but growth is greatly inhibited at 16% salinity and 27°C for the freshwater strain and 16% and 37°C for the brackish water strain [6]. As global temperatures continue to rise, the intrusion of saltwater into freshwater sources will continue to increase *Microcystis* and other MC-producing cyanobacteria, causing them to evolve to be more tolerant of higher salt concentrations.

The microcystin synthetase *mcyA-J* genes make up a 60 kb gene cluster coding for ten proteins that include mixed polyketide synthases (PKSs), non-ribosomal peptide synthetases (NRPSs), and tailoring enzymes [8,11]. These proteins are involved in the addition of different amino acids (Figure 1), the synthesis of the Adda group, and the transport of MC across the membrane [8]. While NRPS creates the backbone of MCs from amino acids, tailoring enzymes modify the peptide chain during or after it is synthesized. For example, *mcyJ* codes for a methyltransferase tailoring enzyme that methylates the Adda group’s fifth carbon [12-14]. The *mcyA–J* gene is divided into two operons with a bidirectional promotor between the two [11]. Environmental factors can regulate the expression of the *mcyA-J* genes, with the *mcyB* gene being regulated by temperature, nitrogen levels, and acetylsalicylic acid levels [8]. The purpose for which cyanobacteria produce microcystin is not completely understood. However, many studies suggest it protects the cyanobacteria from oxidative stress by balancing redox reactions of the electron transport chain [9,10]. A study by Martin *et al.* demonstrated that lower temperatures increase the mRNA expression of *mycA-J* genes and inhibit mechanisms responsible for repairing photosystem II causing dysfunction of the electron transport chain [10]. They concluded that MC is expressed to regulate oxidative stress [10]. Additionally, a study by Stark *et al.* measured the reactive oxygen species (ROS) in both wild-type and *mycA-J* KO in *Microcystis* and determined that knocking out the *mycA-J* gene led to an increase in oxidative stress [9].

## Routes of Human Exposure for MC

Crops are exposed to MC through contaminated irrigation water, in which the MC seeps into the soil, where the plant absorbs it through its roots and distributes it to the edible parts of the plant [2,15]. Additionally, the use of cyanobacteria in fertilizer and compost can add MC to the soil, thus increasing the accumulation in crops [15]. Noteworthy, MC in the soil can impair microorganisms involved in nitrogen fixation, further increasing the bioaccumulation of MC in crops [15]. Not all plants bioaccumulate MC the same way. Aqueous plants bioaccumulate MC by absorbing it from the water through the stoma of their leaves [15]. Leafy vegetables, lettuce, and spinach, as well as other vegetables, such as carrots, tomatoes, and cucumbers, tend to bioaccumulate more MC than fruits [15,16]. A study by Hessal *et al.* demonstrated that the bioaccumulation of MC in basil was proportional to the concentration of MC it was exposed to [17]. Interestingly, it found that more MC accumulated in the roots than in the leaves; however, more was dispelled from the roots than from the leaves [17]. As a result, the basil could still contain MC by the time it is consumed by humans.

Humans are not only exposed to MCs through their food source and drinking water, but they encounter them through their everyday environment, such as exposure to bodies of water and the atmosphere [15]. Many people enjoy recreation around bodies of water where they can be exposed to MC through different means, including accidental ingestion, skin exposure while swimming, and breathing in MC from the air. When MC is ingested, whether it’s through drinking water or a food source in which MC has bioaccumulated, it can be absorbed in the small intestines via transportation through the organic anion transporting polypeptides, OATP3A1 and OATP4A1, in the epithelium cells where it can enter the blood and spread throughout the body [18].

MC can be aerosolized in bodies of water to which humans may be exposed via inhalation [19]. Air becomes trapped under the water as waves break, and aerosolized MC particles are released when bubbles break on the surface of the water [3]. The aerosolized particles containing MC are 2.5 um in size, allowing them to be easily deposited in the lungs [3]. MC directly affects the lungs by altering the gene expression of proinflammatory genes [3].

Dermal exposure can result from being submerged in MC-contaminated water; however, little research has been done on the effect of dermal exposure to MC [20]. MC’s toxic effects most likely remain locally, with the major symptom being skin irritation [20]. No evidence has indicated that MC can be absorbed through the skin into the bloodstream [20]. A study with human epidermal skin cells found that the cells only absorb MC at a very high concentration (10 μM) and only after 48 hours of incubation [21].

## MC and Cytotoxicity

Once transported into the cell by OATP proteins [19], MC can affect many different types of organs (such as lungs, liver, and ovary) and cells (hepatocytes, neurons, and epithelial cells). MC has the most substantial effect on the liver because liver cells express more OATP proteins than other cell types [19]. Therefore, the liver is the most widely studied organ in MC-related cytotoxicity [22]. MC’s major toxic effect is due to its ability to inhibit serine/threonine protein phosphatases PP1 and PP2A [19]. The Adda group orients the MC to the binding site of the phosphatase, and then the MC forms a covalent bond with the binding site [23]. Protein phosphatases PP1 and PP2A enzymes are involved in many signaling pathways, such as AKT, Ras, Src, mTOR, and Wnt/βCatenin [24], which can be dysregulated by MC. Protein phosphatases PP1 and PP2A are involved in regulating the cell cycle by controlling the cell’s entrance and exit from mitosis [25]. Protein phosphatase PP2A regulates the growth and survival of cells by regulating AKT and Ras [24]. Protein phosphatase PP2A is known to be a tumor suppressor, and its inhibition likely leads to cancer through these pathways [19]. Additionally, the AKT pathway regulates the release of proinflammatory cytokines, which can initiate apoptosis. A study by Freytag *et al.* showed that inhibition of Protein phosphatase PP2A can lead to the accumulation of reactive oxygen species (ROS), causing oxidative stress in the mitochondria of *Arabidopsis* [26]. Even though the effect of Protein phosphatase PP2A inhibition specifically on ROS has not been shown in animal models or animal cell cultures, MC has been shown to increase ROS in the mitochondria, leading to apoptosis in different cell types [19].

MC causes mitochondrial dysfunction by damaging mitochondria structure, disrupting metabolism, and damaging mitochondrial DNA [27]. Feng *et al.* showed that MC-LR increases ROS production as well as changes in cell morphology in HepG2 cells depending on dose and exposure time [28]. Similarly, a study by Zhu *et al.* showed that MC-LR causes mitochondrial dysfunction in ovarian granulosa cells by increasing ROS production and inhibiting glucose transporters [29]. Furthermore, MC causes ER stress by disrupting protein folding, increasing ROS production, and disrupting calcium influx [27]. A study by Liu et al. showed that ER stress caused by MC leads to the activation of DNA-damage inducible transcript 3 (Ddit3), which induces apoptosis in mice ovaries [30]. MC can also alter the nucleus and cause chromosomal DNA damage [27].

MC not only causes hepatotoxicity but also affects neuronal cells. Zhang *et al.* showed that MC-LR caused mitochondrial dysfunction in hippocampal neurons in mice and cell culture by increasing the rate of mitochondrial fission, thus causing an accumulation of ROS. MC-LR caused activation of the AKT and calcium/calmodulin-dependent protein kinase II (CaMKII) pathways that regulate mitochondrial fission. As a result, ROS increased, and mitochondrial membrane potential (MMP) and ATP decreased in the mitochondria. Additionally, the MC-LR exposed mice had an altered morphology of mitochondria as well as mitochondria fragmentation in their hippocampal neurons (Table 1) [31]. The mitochondrial dysfunction led to the release of proinflammatory cytokines, which led to the apoptosis of the neurons [31]. A study by Wang *et al.* using mice found that MC-LR caused damage to mitochondrial-DNA (mt-DNA) in the hippocampus and cerebral cortex. A low concentration of MC (1, 5, 10 μg/L in drinking water) caused a slight decrease in the copy number of mt-DNA in the hippocampus, but a high concentration of MC (20 and 40 μg/L in drinking water) caused an increase in copy number of mt-DNA in cerebral cortex most likely due to the mitochondria compensating for high levels of oxidative stress [32]. Additionally, higher levels of ATP6, COX3, and CYTB protein genes coded on mt-DNA involved in oxidative phosphorylation were found in mice exposed to high levels of MC. Furthermore, mice exposed to 20 μg/L and 40 μg/L of MC showed altered morphologies of neurons in the cornu ammonis 1 (CA1), cornu ammonis 3 (CA3), and dentate gyrus (DG) regions of the hippocampus [32]. Xue *et al.* found that MC-LR increased the proliferation of astrocytes by Hippo signaling, a pathway that regulates cell division and differentiation (Table 1). They found that increasing MC-LR up to 0.1 μM increased astrocyte proliferation; however, concentrations of MC over 0.1 μM decreased astrocyte proliferation. At 0.1 μM MC-LR, the Yes-Associated Protein (YAP) translocated into the nucleus, where it activated the transcription factors, epidermal growth factor receptor, and connective tissue growth factor, thereby increasing the proliferation of astrocytes [33].

## MC Disrupts Blood Brain Barrier

The blood-brain barrier (BBB) is a tight barrier of endothelial cells held together by tight junctions and adherens junctions, which are supported by pericytes and astrocytes that regulate the flow of molecules to the brain by allowing nutrients and oxygen to enter but restricting the flow of toxic molecules [34]. The endothelial cells contain various efflux transporters that pump toxic molecules into the circulatory system and drug-metabolizing enzymes to degrade toxic molecules [35]. The tightjunctions are held together by claudin, occludin, and junction adhesion molecules, whereas adherens junctions are held together by cadherin, which is attached to actin filaments by accessory proteins [35]. Pericytes connect closely to the endothelial cells and provide stability to the BBB, control the blood flow through the BBB, and clear toxic molecules [35]. Astrocytes maintain homeostasis in the BBB by regulating the pH and influx of ions [35].

Wang *et al.* studied whether MC-LR could cross and disrupt the BBB in mice and found that MC-LR can enter the cortex and hippocampus of the brain [4]. Evan’s blue dye was used to assess the integrity of the BBB, and they found that MC-LR increased BBB permeability. They determined that the damage of the BBB was caused by the decrease in the occludin responsible for holding the tight junctions together [4]. Additionally, MC-LR increased the expression of matrix metalloproteinases (MMP), indicating further impairment of the BBB’s integrity. Noteworthy, they found that MC-LR caused an increase in microglial activation and immune response in the brain. Together, these results demonstrate that MC-LR can pass the BBB and weaken its integrity, making it more permeable to other toxins by reducing occludin [4].

## MC and Alzheimer’s Pathology

Alzheimer’s disease, the most common form of dementia, is characterized by progressive decline of memory and cognition. Alzheimer’s disease is characterized by the accumulation of β-amyloid plaques and tau neurofibrillary tangles (NFTs), leading to neuroinflammation and, ultimately, neuron death. The amyloid precursor protein (APP) is first cleaved by β-secretase (BACE), followed by γ-secretase, resulting in the production of β-amyloid fragments that can aggregate to form insoluble plaques [36]. β-amyloid increases the phosphorylation of tau by upregulating the kinases GSK-3β and CDK-5 [37]. Hyperphosphorylated tau proteins dissociate from microtubules and form NFTs, which activate astrocytes and microglia, leading to the release of proinflammatory cytokines, neuron death, and cognitive decline [37]. Wang *et al.* showed that MC-LR increased the accumulation of β-amyloid and tau protein phosphorylation in the hippocampus and reduced neuron count in the CA1 and CA3 regions of the hippocampus. Additionally, MC-LR inhibited brain-derived neurotrophic factor (BDNF), a protein that promotes cell proliferation and prevents apoptosis [38]. As a result, mice treated with MC-LR demonstrated impaired spatial memory in the Morris water maze [38]. Zhang *et al.* studied how MC-LR caused tau protein hyperphosphorylation in SH-SY5Y cells and male Sprague Dawley rats. They showed that SH-SY5Y cells uptake MC-LR when incubated with endo-porter peptides. They found that MC-LR causes Protein phosphatase PPA2 subunit C to be demethylated, switching it to its inactive form, thus preventing it from dephosphorylating GSK-3β. Consequently, the activity of GSK-3β increases, resulting in hyperphosphorylation of tau protein. The Protein phosphatase PPA2 regulatory subunit Bα and the catalytic subunit dissociates from the rest of the protein, preventing it from dephosphorylating tau. The hyperphosphorylated tau protein can then dissociate from the microtubule and form NFTs. Like Wang *et al.*’s study, they showed in male rats that the MC-LR accumulates in the hippocampus of the brain, and using the Morris water maze, they demonstrated that MC-LR causes spatial memory impairment [39]. Moreover, Zhang *et al.* showed that metformin could reverse the hyperphosphorylation of tau protein by downregulating mTOR, resulting in the activation of Protein phosphatase PP2A. Additionally, metformin decreases the phosphorylation of GSK-3β, which prevents phosphorylating tau protein. As a result, metformin restored the spatial memory of rats treated with MC-LR. Therefore, metformin could be an effective treatment for MC-LR-induced neurotoxicity [40]. Similarly, a study by Ma *et al.* found that MC-LR treatment of BALB/c mice increased the accumulation of β-amyloid and phosphorylation tau protein in the cortex and hippocampus with the most significant increase at 15 μg/L and 30 μg/L. Additionally, they found that MC-LR treatment increased the mRNA expression of APP and BACE1, the enzyme that cleaves APP. As a result, they observed an increase in the proinflammatory cytokines, Iba-1, caspase-1, IL-1β, and IL-6, along with a decrease in cell viability [41].

Together, these studies show that MC uses its ability to inhibit serine/threonine protein phosphatases PP1 and PP2A to accelerate Alzheimer’s disease pathology, increasing the phosphorylation of key enzymes as well as tau protein itself to increase the accumulation of β-amyloid plaques and Tau NFTs (Table 1). This accumulation then increases the release of proinflammatory cytokines and the activation of apoptosis pathways [19,38,39,41]. The gradual loss of neurons, especially in the hippocampus, leads to the progressive loss of memory seen in Alzheimer’s disease. Through understanding the mechanism in which MC exacerbates Alzheimer’s disease pathology, we can understand how the environments in which people live affect their risk for Alzheimer’s disease and what health disparities exist based on geographic location.

## MC and Parkinson’s Pathology

Parkinson’s Disease (PD) is a neurodegenerative disease that results in a loss of motor ability, tremors, and cognitive decline [42]. It is the result of the loss of dopaminergic neurons primarily in the substantia nigra, a structure of the basal ganglia, caused by the accumulation of α-Synuclein which aggregates into plaques called Lewy bodies, similar to how β-amyloid forms plaques in Alzheimer’s disease [42,43]. Synphilin-1 increases the inclusion formation by α-Synuclein into Lewy bodies by colocalizing with it in the brain and preventing its degradation by proteasomes [42,44]. Conversely, Septin-4 decreases the inclusion formation of α-Synuclein into Lewy bodies [42]. These Lewy bodies can be transferred through the synapse to other neurons, allowing them to spread throughout the brain. Like Alzheimer’s disease, Parkinson’s disease pathology involves mitochondrial dysfunction, microglia activation, and the release of proinflammatory cytokines that lead to the death of neurons [42].

In a study by Yan *et al.*, mice were exposed to MC-LR for 15 months, their brains were studied for Parkinson’s pathology, and their behavior was tested [45]. They found that 7.5 μg/L and 15 μg/L of MC-LR decreased the number of dopamine neurons by 28% and 37%, respectively. Additionally, both 7.5 μg/L and 15 μg/L of MC-LR accelerated apoptosis through the increase in apoptotic proteins Bax, Bcl2, and Caspase-3. The amount of α-Synuclein in the dopamine neuron only increased with the 15 μg/L of MC-LR treatment. However, MC-LR at all three concentrations increased Synphilin-1, the protein that promotes Lewy body formation, and decreased septin-4, the protein that prevents Lewy body formation [45]. The 15 ug/L of MC-LR treatment activated microglia slightly and astrocytes significantly, which resulted in the release of the proinflammatory cytokines TNF-α, IL-6, and MCP-1. Malondialdehyde (MDA), a marker of oxidative stress, increased by 75%, and Superoxide Dismutase (SOD), Catalase, and Glutathione peroxidase (GSH-PX) decreased by 23%, 22%, and 15%, respectively, when exposed to 15 μg/L of MC-LR indicating that MC-LR causes oxidative stress [45]. Mice exposed to the 7.5 μg/L and 15 μg/L of MC-LR had shorter swim times and hang times and took longer to climb than the control mice, indicating that MC-LR impairs motor ability [46]. Another study by Yan *et al.* determined that MC-LR impairs the ubiquitin-proteasome system, which is responsible for degrading α-Synuclein in neurons. MC-LR downregulated the main enzymes of the ubiquitin-proteasome system, UBE1, Parkin, UCHL-1, and ubiquitin. By culturing MC-LR-free microglia cells with neuronal cells containing MC-LR, they showed that MC-LR could be transferred from neuronal cells to microglia and switch microglia to their reactive phenotype. Like their previous study, this resulted in the release of proinflammatory cytokines [45]. Together, both studies show that MC-LR can lead to an increase in Parkinson’s disease pathology by increasing the accumulation of α-Synuclein and decreasing the number of dopamine neurons. It has been shown that MC-LR increases mitochondrial dysfunction and neuroinflammation, which occur in both Alzheimer’s disease and Parkinson’s disease.

## Summary of the Neurotoxic Effect of Cyanotoxin and Current and Future Studies Need to Bridge the Gap

**Figure 2.**
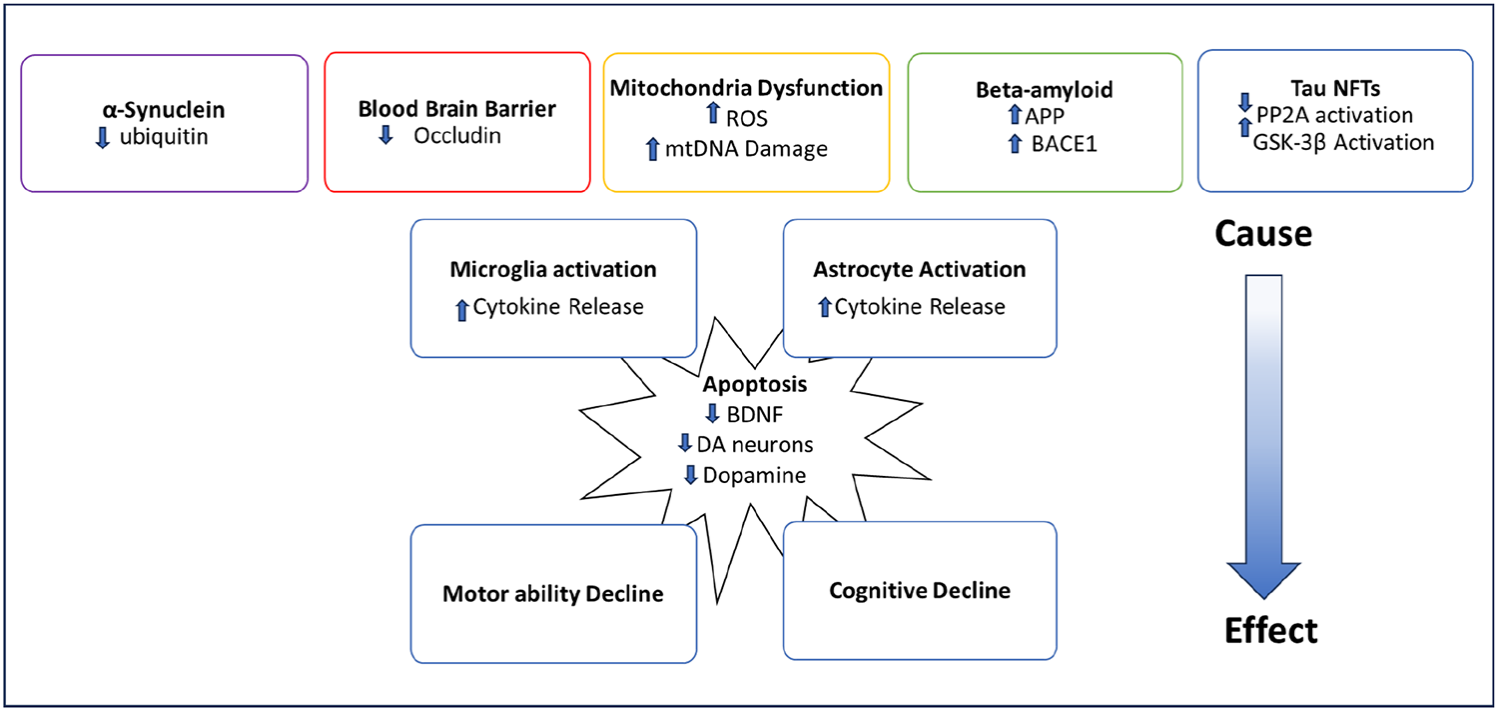
A schematic of how MCs cause neurodegenerative diseases.

The interest in MCs is rising as the climate warms because higher temperatures are leading to an increase in growth from cyanobacteria and, consequently, an increase in MC production. MC is especially a concern in counties with limited access to clean water and where many residents drink untreated water. Additionally, people can be exposed to MC by eating fish and seafood that have bioaccumulated MC, as well as crops that have been irrigated with MC-contaminated water. Since protein phosphatases PP1 and PP2A are present in all cells and vital for many different cellular processes, MC has toxic effects on most organ systems in the body. The brain is especially a concern because MC has been shown to cross the BBB and cause neurodegenerative pathology (Figure 2). Most neurodegenerative diseases are associated with genetic and environmental factors. Improving the living environment can be an effective way of reducing the risk of developing neurodegenerative disease.

Alzheimer’s disease and Parkinson’s disease have been the most studied neurodegenerative diseases in the context of MC-mediated neurotoxicity. However, other forms of MC-induced neuropathology have not been studied as extensively. Lewy body dementia and Parkinson’s disease have similar pathologies caused by the accumulation of α-Synuclein [47]. Studies have shown that MC increases the amount of α-Synuclein in dopamine neurons [46]. Therefore, it is likely that MC increases the risk of Lewy body disease by the same mechanism in which it accelerates Parkinson’s disease pathology (Figure 2).

MC’s effect on neurodegenerative disease has been studied primarily in wild-type animal models, so MC’s effect in conjunction with certain genetic risk factors is yet to be studied. Therefore, MC might have varying effects on different people based on the diversity of the gene pool and single nucleotide polymorphisms (SNPs). For example, APOE4 is the primary genetic risk factor for Alzheimer’s disease, but its interaction with environmental factors like MCs has not yet been investigated. The APOE4 allele is known to increase mitochondrial dysfunction and endoplasmic reticulum (ER) stress, like the effects of MCs [48,49]. Therefore, it is probable that APOE4 exacerbates MC’s effect on mitochondrial dysfunction and ER stress in people with this genotype.

Further, MC has been studied extensively in cell culture and animal models as a stand-alone toxin; however, the effect of MC in combination with alcohol has not been studied. Both MC and alcohol lead to the accumulation of ROS in neurons, resulting in mitochondrial dysfunction [50]. Additionally, both MC and alcohol cause the activation of microglia cells, resulting in the release of proinflammatory cytokines with both MC and alcohol, increasing levels of TNF-α, IL-6, and MCP-1 in the brain [46,50,51]. As a result, both MC and alcohol increase the risk for Alzheimer’s disease and likely have an additive effect on its pathology.

There are at least 279 variations of microcystin, with MC-LR being the most widely studied; however, research on other MCs besides MC-LR is limited. More research is needed to understand the effects all these different variations of MC have on the brain and to combat the threat that MC poses to human health. By understanding the mechanisms of MC toxicity, new therapeutics can be developed to prevent and treat neurological damage caused by MC. Metformin is a feasible treatment against MC-induced neurotoxicity, and more already-developed drugs are likely to be effective at protecting the brain from MC [40]. Emerging research on how MC affects the brain could lead to a better understanding of how the environment impacts brain health.

## Conclusions

Cyanotoxins (CTs) are major emerging toxins released by photosynthetic cyanobacteria (algal blooms). Global warming and alternations of temperature in aquatic systems increased the biomass of algal bloom that released CTs predominantly. Direct or indirect exposure to CT causes several human health-related issues. Although CTs primarily target the liver, induce hepatotoxicity, and modulate liver diseases, CTs can also pass BBB and exert neuronal cytotoxicity. In this review, we summarized the presence of cyanobacteria genus that potentially releases MC in aquatic systems. We also discussed possible MC bioaccumulation in humans through the food chain. Notably, we evaluated the molecular mechanisms associated with neurotoxicity, AD, and PD signaling modulated by MC. However, more efforts are required for MC exposure and risk assessments since different variations of MC are reported. By understanding the mechanisms of MC toxicity, new therapeutics can be developed to prevent and treat neurological damage caused by MC.

## Figures and Tables

**Table 1.** Summary of Animal Model, Brain Regions affected, and Cellular Mechanism of MC-induced neurodegenerative diseases.

Animal/Cell Model	Affected Brain Region	Mechanism	Reference
BALB/c Mice and neuronal cell culture	Hippocampus	↑ Mitochondrial Dysfunction ↑ ROS ↑ AKT and CaMKII ↑ Cytokine release ↑ Apoptosis	[28]
C57BL/6 mice	Hippocampus (CA1, CA3, DG) (greatest effect) cerebral cortex	↑ Neuron morphology changes ↑ mt-DNA damage	[29]
BALB/c mice and primary astrocyte culture	cerebral cortex Hippocampus (DG)	↑ Astrocyte proliferation ↑ Hippo signaling ↑ Cytokines ↑ ROS	[30]
BALB/C mice and hCMEC/D3 and BV-2 microglial cell culture	hypothalamus cortex hippocampus	↓ BBB integrity ↓ Occludin ↑ Microglia activation	[4]
BALB/c mice	Hippocampus (CA1, CA3)	↓ Neurons numbers ↑ β-amyloid ↑ Tau ↓ BDNF ↓ Spatial memory	[35]
Male Sprague Dawley rats and SH-SY5Y cells	Hippocampus	↓ PP2A activation ↑ GSK-3β activation ↑ P-Tau ↓ Spatial memory	[36]
BALB/c mice and HT-22	Hippocampus	↑ β-amyloid ↑ Tau ↑ APP & BACE1 ↓ Cell viability	[38]
Male BALB/c mice and SH-SY5Y cells	Cortex Hippocampus Substantia Nigra	↑ α-Synuclein ↓ DA neuron numbers ↓ Dopamine ↑ Cytokines ↓ Motor ability	[43]
SH-SY5Y cells and HMC3 cells	Cell culture only	↑ α- Synuclein ↓ Ubiquitin ↑ Reactive microglia ↑ Cytokines	[44]

## Data Availability

All data generated are included in this manuscript and available upon the request to the corresponding author.
